# Theta-alpha cross-frequency synchronization facilitates working memory control – a modeling study

**DOI:** 10.1186/2193-1801-2-14

**Published:** 2013-01-17

**Authors:** David Chik

**Affiliations:** Department of Brain Science and Engineering, Kyushu Institute of Technology, Kyushu, Japan

**Keywords:** Working memory, Synchronization, Cross-frequency

## Abstract

Despite decades of research, the neural mechanism of central executive and working memory is still unclear. In this paper, we propose a new neural network model for the real-time control of working memory. The key idea is to consider separately the role of neural activation from that of oscillatory phase. Neural populations encoding different information would not confuse each other when the populations have different oscillatory phases. Depending on the current situation, relevant memories bind together through phase-locking between theta-frequency oscillation of a Central Unit and alpha-frequency oscillations of the relevant group of Memory Units. The Central Unit dynamically controls which Memory Units should be synchronized (and the encoded memory would be processed), and which units should be out of phase (the encoded memory is standby and would not be processed yet). Simulations of two working memory tasks are provided as examples. The model is in agreement with many recent experimental results of human scalp EEG analysis, which reported observations of neural synchronization and cross-frequency coupling during working memory tasks. This model offers a possible explanation of the underlying mechanism for these experiments.

## Introduction

According to the classical theory of psychology, the working memory system can be thought of having a star-like architecture with a central element and several peripheral elements (Baddeley & Hitch, 1974; Baddeley, (1992). The central element is known as “central executive”, which controls how to select and process information. Peripheral elements are buffers for short-term storage of small amount of information (for example, a phonological loop for auditory information and a visuo-spatial sketch pad for visual information). Despite decades of research, however, it is not clear how auditory, visual, and other information, which are stored at different locations, can be coordinated in order to provide a coherent cognitive function.

Based on recent EEG and fMRI studies, prefrontal cortex has been considered to be one possible candidate for central executive function because of its critical role in top-down modulation D’Esposito, (2007). Prefrontal cortex plays an active role in the shift of attention Rossi et al., (2009) and task switching Hyafil et al., (2009). It can also bias its effective connectivity towards different posterior visual areas depending on the domain of visual features to which the subject attended Morishima et al., (2009). In visual working memory task, activity in the prefrontal cortex and basal ganglia preceded the filtering of irrelevant information McNab and Klingberg, (2008). Based on these findings, it becomes a very interesting question to ask, what is the neural mechanism for prefrontal cortex to coordinate with posterior areas (e.g. visual cortex and auditory cortex) during working memory tasks?

The temporal correlation hypothesis has been proposed to be the mechanism of binding. Classically, this hypothesis was limited to the binding of various visual features of an object Treisman & Gelade, (1980); Singer, (1999); Von der Malsburg, (2001). But recent experiments suggest a more powerful role of neural coherence. The coherence of spikes plays a major role in the control of attention e.g. Steinmetz et al., (2000); Fries et al., (2002), perception e.g. Nakatani & van Leeuwen, (2006); Melloni et al., (2007), and memory e.g. Klimesch et al., (2008); Anderson et al. (2010). Abnormal neural oscillations and synchrony have been observed in patients of psychiatric disorders e.g. Brown, (2005); Uhlhaas & Singer, (2010).

Recently, Kawasaki et al. (2010) reported that during human visual and auditory working memory tasks, they observed alpha rhythm activities of around 12 Hz in sensory specific regions (i.e. parietal or temporal cortex) which may associate with memory storage. They also observe theta rhythm of around 6 Hz in frontal region during manipulation period. A similar study by Sauseng et al. (2009) showed that cross-frequency phase synchronization between theta and gamma oscillations at parietal regions is associated with successful maintenance of relevant material in short term memory. Also, alpha activity increases with memory load. Other studies also showed that different items in working memory are supported by different synchronized neural oscillations at high frequency from alpha to gamma range. These activities are located at different phases of a background oscillation of a lower frequency in theta range. Cross-frequency coupling between the two oscillations has been observed Jensen & Lisman, (2005); Siegel et al., (2009); Axmacher et al., (2010).

Although there is disagreement on exactly which frequency bands concern with what function, the above evidences converge to a theory that, cross-frequency coupling and phase coding may serve as an important neural mechanism underlying the working memory process (for a recent review, see Fell & Axmacher, (2011). However, what exactly is the association between the above experimental observations and the implementation of working memory? In this paper, we use model simulation to illustrate how a neural network can generate cross-frequency, dynamical synchronization for a selected group of neural units, and how it can carry out the operation and control of working memory.

## Model implementation

### Working memory tasks

**Figure 1:**
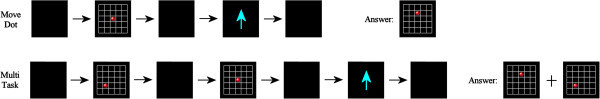
**Working memory tasks being used in our simulation. (a)** Move-a-dot task: move the position of a red dot according to the direction of an arrow. **(b)** Multi-task: memorize the position of another red dot while doing a move-a-dot task.

We shall consider two working memory tasks. The first task is called Move-a-dot, which is shown in Figure 1a. The screen would show briefly a visual signal of a red dot inside a grid, followed by a blank screen. After that, the screen would show another visual signal which is an arrow. The subject would need to move the position of the dot according to the direction of the arrow.

The second task is called Multi-task, which is shown in Figure 1b. The screen would show a red dot inside a grid, followed by another red dot and an arrow. The subject would need to memorize the position of the first dot, and then do a move-a-dot task using the second and third visual signal.

### Neural network structure

**Figure 2:**
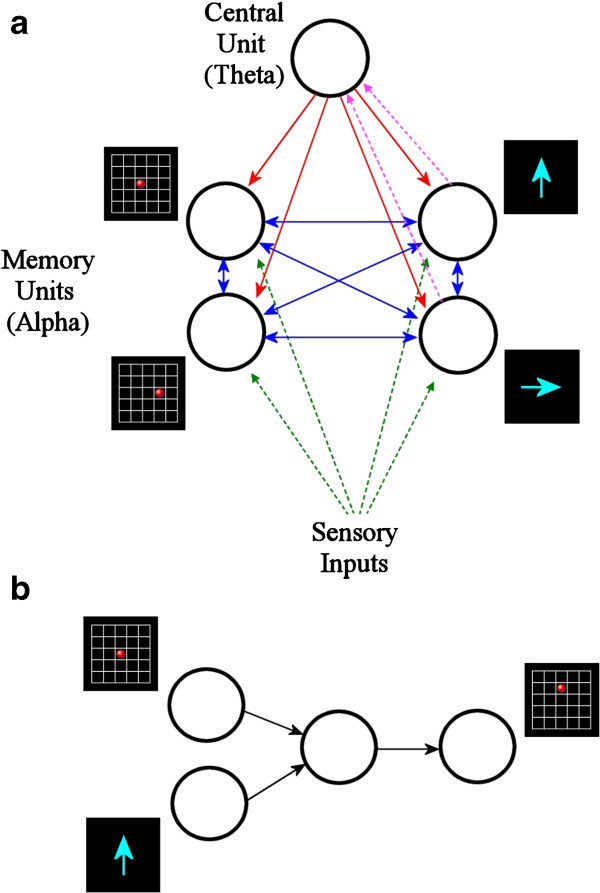
**Network architecture.** Neural units are represented by circles. **(a)** The executive control network consists of one Central Unit and some Memory Units which represent different positions of a dot or different directions of an arrow. The Central Unit oscillates at theta rhythm while the Memory Units oscillate at alpha rhythm. The Central Unit tries to synchronize the Memory Units (red arrows) but the Memory Units de-synchronize each other (blue arrows). Memory Units receive sensory inputs (green arrows). Motion-related Memory Units can give a go / no-go signal to the Central Unit (pink arrows). **(b)** One position-related Memory Unit and one motion-related Memory Unit converge to a co-incidence detection unit which generates the output by activating the corresponding position-related Memory Unit.

Let us consider a neural network with convergent-style (or star-like) architecture, as shown in Figure 2a. This type of network with a central element has been used for modeling selective attention Borisyuk & Kazanovich, (2003); Chik et al., (2009), novelty detection Borisyuk & Kazanovich, (2004), image object segmentation Wang & Terman, (1995); Borisyuk et al., (2009a), as well as visual perception of ambiguous figures Borisyuk et al., (2009b). In the present study, we borrow this modeling idea to consider a working memory system consisting of a Central Unit and some Memory Units. The Central Unit connects to all Memory Units through synchronizing connections (shown as red arrows in Figure 2a). The Memory Units connect to each other through de-synchronizing connections (blue arrows in Figure 2a). In addition, the system is controlled by two currents: First, sensory inputs (green arrows in Figure 2a), which, in our case, are the visual signals for the working memory tasks; Second, executive signal (pink arrows in Figure 2a), which is a “go” or “no-go” signal being delivered to the Central Unit from a motion cue.

In terms of physiology, the Central Unit represents the central executive which is responsible for the manipulation of working memory. It is a population of neurons probably located in prefrontal cortex, and we are interested in its theta frequency oscillation due to aforementioned experimental results. In addition, we assume that there are some neurons which respond selectively to various stimuli. These neurons may be located in parietal cortex or frontal eye field. We called them Memory Units which represent the memory of either various positions of a red dot in a grid, or various motion commands from arrow symbols. We assume that their activity is in the alpha band. In terms of physiology, the neurons encoding the location of a red dot may be found along the dorsal stream Mishkin & Ungerleider, (1982) while the neurons recognizing the arrow symbols may be found in the ventral stream. Therefore, the memory units may be located in different brain regions. We wish to remark that in real brain, the connectivity between dorsal and ventral visual streams is extremely complicated. The model here only reflects a portion of the actual network. We assume that this portion of network helps to carry out the executive function, which will be demonstrated later. In addition, we also remark that we do not propose grandmother cell. We assume one memory unit corresponds to one visual signal just for the sake of simplicity.

Generation of output is given by another network as shown in Figure 2b. One position-related Memory Unit and one motion-related Memory Unit converge to a coincidence detection unit which can activate the corresponding position-related Memory Unit as an output. The correct mappings between inputs and outputs have been trained beforehand.

### Equations

There are many options for our model simulation, such as using Hopfield model Hopfield, (1982), phase oscillator model Kuramoto, (1984; Ermentrout, (1994), or conductance-based neuronal models. The author decides to use Wilson-Cowan oscillator model Wilson & Cowan, (1972); Wilson, (1999) because this model is simple yet sufficient for the study of both activity and oscillatory phase. Let us consider a neural “unit” which consists of a group of excitatory and inhibitory neurons. The activity rate of this unit is,123

where *E*(t) is the activity rate of the excitatory neurons inside this unit; and *I*(t) is the activity rate of the inhibitory neurons inside this unit. The values of parameters are:*a*_1_=0.26;*a*_*2*_*=*0.13;*b*_1_=1.6;b_2_=1.5;c_1_=100;c_2_=30. The values of*a*_1_ and *a*_2_ are used for adjusting the time scale to the range of theta to alpha frequency band. The values of (*b*_1_*b*_2_*c*_1_*c*_2_) are identical to those given by Wilson (1999).

External current received by the unit is denoted by *K*(t). Regarding the Central Unit, *Kc*(t)=0 or 5 corresponding to whether the Central Unit is quiet or activated. Regarding the Memory Unit, this term incorporates the effect of neural connections:4

Where *K*_0_=0 or 20 corresponding to whether the Memory Unit is quiet or activated, which is controlled by the visual inputs. The middle term corresponds to a synchronizing influence from the Central Unit to the Memory Units, with connection strength *w*_1_. The right term with a summation corresponds to a de-synchronizing influence among *N* Memory Units, with connection strength *w*_2_. This is not a winner-take-all competition because *w*_2_ is not strong enough to silence an opponent. The role of this term is simply de-synchronizing.

In the simulations, we numerically integrate the equations using fourth-order Runge–Kutta method with a fixed time step equals to 0.01 milliseconds. Reliability of numerical integration is guaranteed by the fact that using different initial values or doubling the time step will not affect the simulation results.

## Results

### Basic dynamics

**Figure 3:**
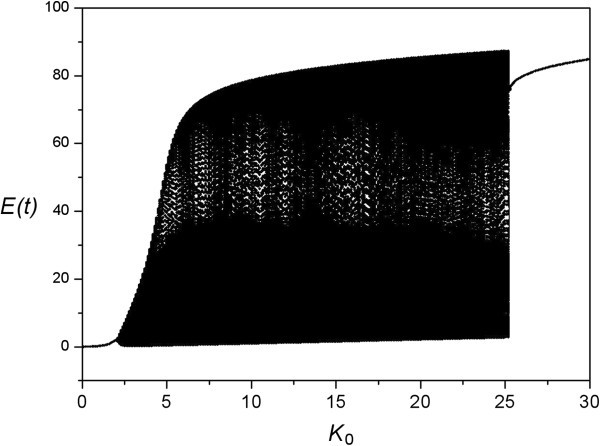
**Bifurcation diagram of a Wilson-Cowan unit as a function of constant current*K***_**0.**_ The dark area in 2<*K*_0_<25 indicates the existence of a stable limit cycle.

First, let us consider a single Memory Unit with no connections to other units, that is, *w*_1_=0;*w*_2_=0. We only change the constant current *K*_0_. As shown in Figure 3, the dynamics of the unit is described by a fixed point when 0 < *K*_0_ < 2; and then a limit cycle when 2 < *K*_0_ < 25; and finally a fixed point again (saturation) when *K*_0_ > 25.

Next, let us consider a network of one Central Unit and 4 Memory Units. The Central Unit receives a constant current of *Kc*=5 and oscillates at theta frequency, while the Memory Units are oscillating at alpha frequency due to a higher constant current *K*_0_=20 We investigate the effect of connections described by the second and third terms in Equation 4.

**Figure 4:**
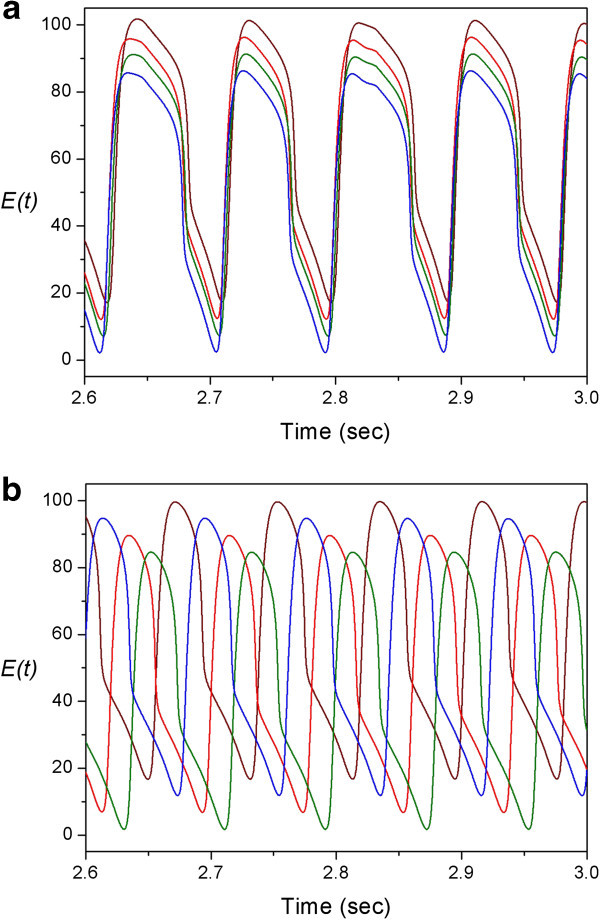
**Activity rates of the excitatory components of 4 Memory Units.** To give a clear distinction, different colors are used for different Memory Units, and also the values of their activity rates are modified as *E*(t);*E*(t)+5;*E*(t)+10;*E*(t)+15 respectively. Parameter values are given by: **(a)***N*=4;*K*_0_=20;*w*_1_=0.1;*w*_2_=0. **(b)***N*=4;*K*_0_=20;*w*_1_=0;*w*_2_=0.02.

The influence from Central Unit (the second term) can be seen by setting *w*_1_=0.1;*w*_2_=0. As shown in Figure 4a, the 4 Memory Units become synchronized. It is interesting to note that their waveforms show some irregularities although there is no noise in the system. Their oscillations are not periodic but quasi-periodic. Therefore, it is actually a complex, high dimensional dynamical behavior.

In addition, the influence among Memory Units (the third term) can be seen by setting *w*_1_=0;*w*_2_=0.02. As shown in Figure 4b, the 4 Memory Units are totally de-synchronized. Their phases distribute evenly along an alpha cycle.

When both influences exist (both *w*_1_ and *w*_2_ are non-zero), we shall observe a very interesting phenomenon where some of the Memory Units are synchronized while others are not, which will be shown in the next section.

### Simulation of move-a-dot working memory task

We perform a simulation of a move-a-dot working memory task, which has been described in Figure 1a. In the task, the model needs to move a dot to a new location according to the direction of an arrow sign. In the simulations, we use *N*=3 Memory Units to represent two positions of dots and one direction of an arrow. The values of connection strength are *w*_1_=0.15;*w*_2_=0.005.

**Figure 5:**
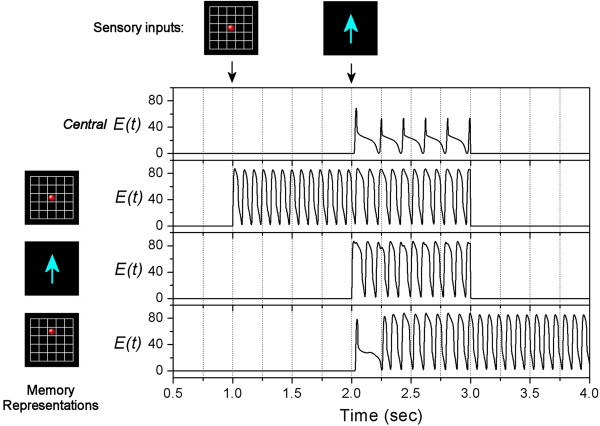
**Simulation of move-a-dot task.** On the top of the figure, it shows the appearance of sensory inputs (visual signals) during the task. The main figure shows the activity rates of the Central Unit (top panel) and 3 Memory Units respectively. The Memory Units represent different visual objects which are shown on the left side.

The activity rates of the units are shown in Figure 5. At the beginning, all units are quiet with zero activity rates. At time = 1 sec, a visual signal of a red dot appears briefly, which activates the corresponding position-related Memory Unit (second panel in Figure 5). The activation is due to the sensory input current of *K*_0_=20 (shown as green arrows in Figure 2a). This Memory Unit keeps oscillating so that the memory is retained. At time = 2 sec, another visual signal of an arrow sign appears briefly, which activates the corresponding motion-related Memory Units (third panel in Figure 5), again by the sensory input current of *K*_0_=20 (shown as green arrows in Figure 2a). Immediately after that, this motion-related Memory Unit sends a “go” signal to activate the Central Unit (top panel in Figure 5) by a current of *Kc*=5 (shown as pink arrows in Figure 2a). Now, the Central Unit tries to synchronize the Memory Units (shown as red arrows in Figure 2a) while the Memory Units try to de-synchronize each other (shown as blue arrows in Figure 2a). In this case, since the synchronizing influence is larger than the de-synchronizing influence (*w*_1_ ≫ *w*_2_), the position-related and motion-related Memory Units are synchronized. Also, the Central Unit forms theta-alpha cross-frequency synchronization with the relevant Memory Units. After that, manipulation is carried out through another network as shown in Figure 2b. The current position-related and motion-related Memory Units connect to a coincidence detection unit with a threshold of 160. When the sum of their activity rates is higher than this threshold, the coincidence unit would deliver a current to the output position-related Memory Unit. We assume that this current increases from 0 to 20 at the rate of 0.1/msec. As a result, the output unit is activated and oscillates at alpha frequency after time = 3 sec (bottom panel in Figure 5). Finally, after manipulation is completed, previous memories are suppressed. The exact mechanism of how the brain decides to forget some memories is beyond the scope of this study. Here we simply assume that at time = 3 sec, the currents *Kc* for Central Unit and *K*_0_ for the previous two Memory Units drop back to zero.

We wish to remark that, without the network structure as shown in Figure 2a, the position-related and motion-related Memory Units activated by sensory inputs may have different initial phases, and therefore the sum of their activity rates may not pass the threshold of the coincidence unit so as to generate the output. Therefore, this network structure is necessary to provide the theta-alpha cross-frequency synchronization for the processing of information.

### Simulation of multi-task working memory operation

**Figure 6:**
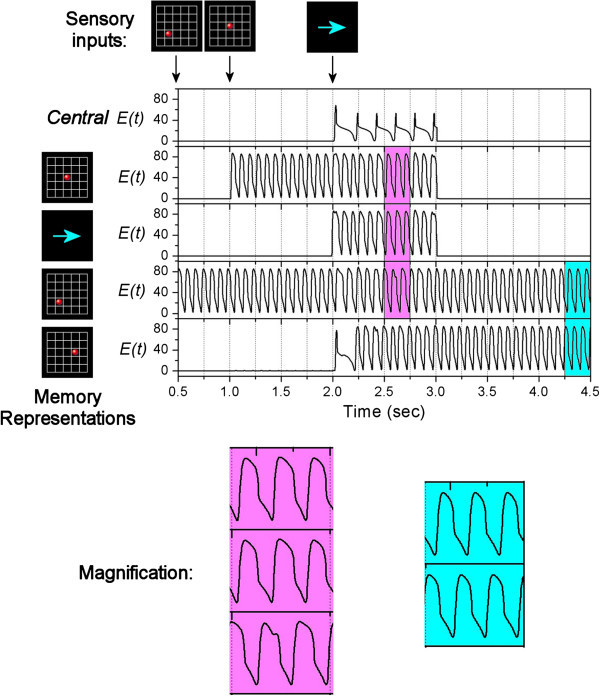
**Simulation of multi-task.** On the top of the figure, it shows the appearance of visual signals during the task. The main figure shows the activity rates of the Central Unit (top panel) and 4 Memory Units respectively. The Memory Units represent different visual objects which are shown on the left side. The pink and blue portions of the main figure are magnified and shown at the bottom.

Let us consider a multi-task operation as described in Figurer 1b. This time, the model needs to perform the same move-a-dot task while in addition, it needs to memorize an extra object during the operation. We use one Central Unit and *N*=4 Memory Units. In the simulation, the values of connection strength are *w*_1_=0.2;*w*_*2*_=0.02. The resulting activity rates of the units are plotted in Figure 6. In order to see their relative phases more clearly, magnifications of the plot are also provided below the main figure.

At time = 0.5 sec, a visual signal of a red dot arrives, which activates a position-related Memory Unit (second bottom panel in Figure 6) by a current of *K*_0_=20 (shown as green arrows in Figure 2a). The model needs to memorize this extra object until the end of task. Apart from that, the subject also needs to do another task. At time = 1 sec, another visual signal appears briefly and activates another position-related Memory Unit (second panel in Figure 6). At time = 2 sec, a visual signal of an arrow appears briefly, which activates a motion-related Memory Unit (third panel in Figure 6). This motion-related unit sends a “go” signal to the Central Unit (top panel in Figure 6) by a current of *Kc*=5 (shown as pink arrows in Figure 2a). The Central Unit tries to synchronize the Memory Units while the Memory Units try to de-synchronize each other. By choosing *w*_1_=0.2;*w*_2_=0.02, the two influences compete with each other. The dynamics settles to a situation where the position-related Memory Unit of the second visual signal synchronizes with the motion-related Memory Unit, while the unit representing the first visual signal is anti-phase compared to the other two Memory Units (pink magnification in Figure 6). As a result, manipulation of working memory occurs. The synchronized position-related and motion-related units converge to a coincidence detection unit (see the network structure in Figure 2b). Again, the coincidence unit has a threshold of 160, and it delivers a current to the output when the sum of the two inputs is higher than this threshold. The output unit is therefore activated (bottom panel of Figure 6). After manipulation is completed (at time = 3 sec), the Central Unit and the previous Memory Units are suppressed. Now, the unit representing the first visual signal and the output unit of the move-a-dot task de-synchronize each other (shown as blue arrows in the network structure in Figure 2a). Hence they become anti-phase (blue magnification in Figure 6). The system successfully performs a multi-task operation by utilizing “phase coding” and “partial synchronization” as a way to distinguish relevant and irrelevant working memories.

### Error correction

**Figure 7:**
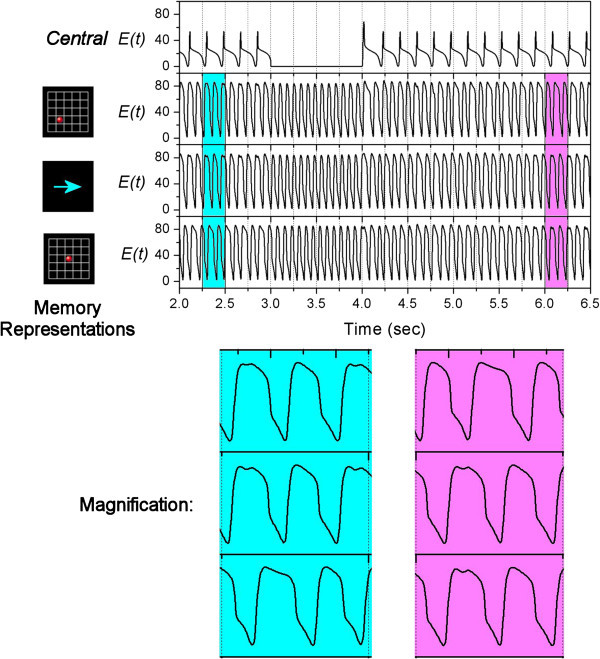
**Error correction.** The main figure shows the activity rates of the Central Unit (top panel) and 3 Memory Units respectively. The Memory Units represent different visual objects which are shown on the left side. The blue and pink portions of the main figure are magnified and shown at the bottom.

The result shown in Figure 6 is sensitive to initial phases of the units and timings of stimulus onset. It is possible that the system makes a mistake by synchronizing the wrong groups of units. In real life, wrong binding of information can happen when people are not paying attention. For example, a busy housewife may bring the clothes to the rubbish bin while taking the rubbish to the laundry. Usually the person would realize the error when the outcome is not right. In our model, there is an error correction mechanism, as shown in Figure 7. In the simulation, we use *N*=3 Memory Units. The values of connection strength are *w*_1_=0.2;*w*_2_=0.02.

At first, there is synchronization between a position-related and a motion-related Memory Units, while there is another position-related Memory Unit which is anti-phase (blue magnification in Figure 7). Assume this binding is wrong. The exact mechanism of error detection by the brain is beyond the scope of this study. Here, our goal is to show that the dynamics of partial synchronization can be adjusted by the Central Unit. At time = 3 sec, the Central Unit is shut down by setting *Kc*=0. As a result, the only force remained in the network is the de-synchronizing influence among the 3 Memory Units. Then at time = 4 sec, the Central Unit is reactivated again by setting *Kc*=5. The system is reconfigured and this time, the motion-related Memory Unit synchronizes with another position-related Memory Unit (pink magnification in Figure 7).

The decision of which units would be synchronized and which would not is based on the timing of the reactivation of the Central Unit. Suppose the Central Unit does not know which units should be bound, the reconfiguration can be considered as totally random. It is possible that the new binding is wrong again (e.g. the two position-related units become synchronized leaving the motion-related unit to be anti-phase). However, since the number of stored objects is limited, there is a high probability of making a correct binding after the reset.

### Three states of memory

As shown in Figures 6 and 7, we can see that in this model, a Memory Unit has 3 different states:

1. Quiet state (zero activity rate), which corresponds to “forgotten” of memory;
2. Active plus synchronized state, which corresponds to the situation when the information is being processed;
3. Active but de-synchronized state, which corresponds to the situation when the information is stored but not processed (“standby” for later use).

### Speed of synchronization

According to Figures 5 and 6, the network takes less than one second to establish the partial synchronization between units from different initial phases to the situation where the error of their relative phase becomes smaller than 0.01 alpha cycles. However, Figure 7 shows that after the reactivation of Central Unit at time = 4 sec, the network takes around 2 seconds to re-establish the partial synchronization. Therefore the synchronization time can range from less than one second to perhaps more than 2 seconds.

### Working memory capacity

In psychology, there is a capacity limit in working memory. Early studies suggested that a maximum of 7 items can be maintained in working memory without causing confusion Miller, (1956), but later studies argued for a smaller capacity of about 4 chunks in young adults, and fewer in children and elderly Cowan, (2001). In our model, it seems that an unlimited number of objects can be stored as standby (de-synchronized state). However, the reliability of information processing would become poor with increasing memory load. This is because the error correction mechanism (Section 3.4) is just a random binding of Memory Units. When more objects are stored, the probability of getting a correct binding would decrease exponentially.

## Discussion

Working memory plays a pivotal role in intelligence, because it involves selection, temporary storage, processing and update of information according to a task. Therefore, understanding the neural mechanism of working memory is very important for us to understand the generation of intelligence and higher cognitive functions. In this paper, we demonstrated the principle of partial, cross-frequency synchronization as one possible mechanism to facilitate working memory control.

### Novelty of this model

Regarding the dynamics of partial synchronization, a model of winner-less competition has been proposed Rabinovich et al., (2008); Ashwin & Lavric, (2010). However, the current model is different from the winner-less competition system. In winner-less system, all groups will have times of synchronization and times of de-synchronization, one after one. In our model, however, synchronization is determined by sensory inputs and controlled by the Central Unit.

Some models of working memory have been proposed before. For example, Brunel and Wang (2001) proposed a cortical network model that can provide stable persistent activity to maintain one item in working memory. Lundqvist et al. (2010) described an attractor neural networks that can generate beta to gamma oscillations. Other models considered a mixture of working memory and long term memory system, and they studied how information is learned and then recalled through modification of connection strengths e.g. O’Reilly et al., (1999); Szatmary & Izhikevich, (2010); Pascanu & Jaeger, (2011). In comparison, our model complements the previous ones by offering the following new insights:

1. **Multi-task.** This model allows dynamical control of multiple memories which may or may not be relevant to a particular task.
2. **Agreement with EEG.** This model allows an association to EEG observations. Alpha synchronization concerns with maintenance of working memory while theta-alpha cross-frequency synchronization concerns with manipulation, which is in agreement with Kawasaki et al. (2010).
3. **Cross-frequency coupling.** The control from Central Unit to Memory Units is not a simple 1:1 synchronization, but a more difficult, cross-frequency coupling. This kind of dynamical control is not common.
4. **Real-time operation.** Synchronization between units is very fast as reported in Section 3.6, so real-time operation of working memory can be achieved.
5. **Agreement with temporal correlation hypothesis.** This is an extension of the traditional temporal correlation hypothesis. The function of partial synchronization is to bind relevant Memory Units according to the task, and resetting the Central Unit can control which group of Memory Units to be synchronized.
6. **Agreement with the psychological theory of memory availability.** Psychological experiments suggest the existence of limited amount of information which is not manipulated but in a “readily accessible state” Peters et al., (2008). In our model this can be represented as “active but de-synchronized state” of a Memory Unit. Besides, our model also agrees with Cowan’s model of working memory Cowan, (1999). In his model, there are 3 states of memory: dummy long term memory, a subset of working long term memory that is currently activated, and the subset of activated memory that is in the focus of attention. These may correspond to the 3 states of memory in our model: quiet, active plus synchronized, active but de-synchronized (see Section 3.5).

### Prediction of this model

Our model provides the following testable prediction: In real life, a person usually needs to process many pieces of information in working memory. During this multi-task situation, neurons corresponding to different tasks should keep being activated. Controlling the phases may be a way to avoid confusion among activated neurons. Siegel (2009) reported that when a subject memorized two visual objects in short term memory, the neural activities fell into two phase values above the background 3 Hz rhythm. Of course his experimental setup was different from the one described in this paper. Hence it will be interesting to see if a future experiment can confirm the following: if a subject needs to do two working memory tasks sequentially (e.g. one is visual and one is verbal), this model predicts that synchronization and resetting of phase values of neural oscillations will be switched between task-relevant brain regions (e.g. switching from a visual task to a verbal task may be reflected from a switch of synchronization from between visual and prefrontal cortices to a different synchronization between auditory and prefrontal cortices.). This experiment can be done by advanced local field potential recordings and tetrode recordings Kucewicz et al., (2011).

### Future works

Future developments of this model include: First, we need to incorporate long term memory. At present, we only consider external stimuli from the environment. The influence from long term memory is not yet considered. In future, this can be implemented by inserting a long term memory module, and introducing a learning mechanism such that some psychological behavior can be reproduced (e.g. neural units representing a similar topic will be easier to synchronize).

Next, we need to revise the error correction system. At present, there is a naïve error correction system which randomly tries out different combinations of synchronized units. This is like a baby uses trial-and-error method to work out the correct answer. However, previous experience (recall of long term memory) should play a role in making the error correction system more efficient.

In addition, we need to consider the complex factors affecting the reliability of working memory. For example, memories of a similar topic may interfere with each other Oberauer & Kliegl, (2006), attention demand may speed up the decay of previously stored working memory Barrouillet et al., (2004), and so on. A detailed, quantitative comparison between the model and these complex psychological evidences on working memory capacity will be the target for future study.
